# Hyperproduction of extracellular polymeric substance in *Pseudomonas fluorescens* for efficient chromium (VI) absorption

**DOI:** 10.1186/s40643-023-00638-3

**Published:** 2023-03-08

**Authors:** Lijie Yang, Zhen Chen, Ying Zhang, Fuping Lu, Yihan Liu, Mingfeng Cao, Ning He

**Affiliations:** 1 Department of Chemical and Biochemical Engineering, College of Chemistry and Chemical Engineering, Xiamen University, Xiamen, 361005 People’s Republic of China; 2 Shandong Institute of Commerce and Technology, Jinan, 251000 People’s Republic of China; 3 College of Life Science, Xinyang Normal University, Xinyang, 464000 People’s Republic of China; 4 Key Laboratory of Industrial Fermentation Microbiology, Ministry of Education, Tianjin Key Laboratory of Industrial Microbiology, The College of Biotechnology, Tianjin University of Science and Technology, Tianjin, 300457 People’s Republic of China; 5 The Key Lab for Synthetic Biotechnology of Xiamen City, Xiamen University, Xiamen, 361005 People’s Republic of China

**Keywords:** Extracellular polymeric substances, *Pseudomonas fluorescens*, ARTP, Chromium, Fermentation

## Abstract

A novel extracellular polymeric substance (EPS) with flocculating activity produced by *Pseudomonas fluorescein* isolated from soil was studied in this paper. Firstly, atmospheric and room temperature plasma (ARTP) was applied to get a mutant of *P. fluorescein* with higher EPS production. A mutant T4-2 exhibited a 106.48% increase in flocculating activity compared to the original strain. The maximum EPS yield from T4-2 was enhanced up to 6.42 g/L, nearly 10 times higher than the original strain on a 3.6-L bioreactor with optimized fermentation conditions. Moreover, the flocculating activity of the mutant reached 3023.4 U/mL, 10.96-fold higher than that of T4. Further identification showed that EPS from mutant T4-2 was mainly composed of polysaccharide (76.67%) and protein (15.8%) with a molecular weight of 1.17 × 10^5^ Da. The EPS showed excellent adsorption capacities of 80.13 mg/g for chromium (VI), which was much higher than many reported adsorbents such as chitosan and cellulose. The adsorption results were described by Langmuir isotherm and pseudo-second-order kinetic model. The thermodynamic parameters (Δ*G*^0^, Δ*H*^0^ and Δ*S*^0^) revealed that the adsorption process was spontaneous and exothermic. Adsorption mechanisms were speculated to be electrostatic interaction, reduction, and chelation.

**Graphical Abstract:**

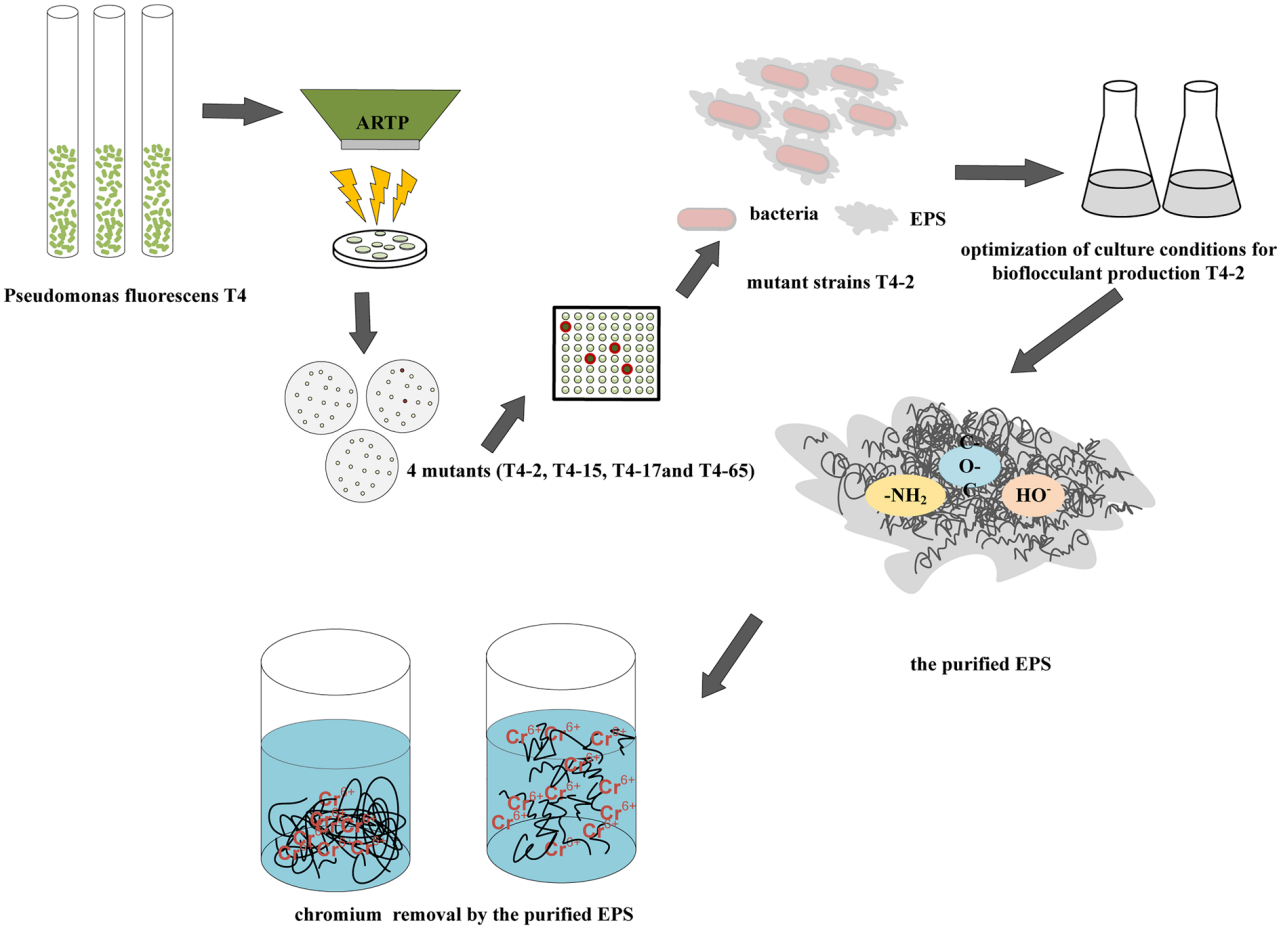

**Supplementary Information:**

The online version contains supplementary material available at 10.1186/s40643-023-00638-3.

## Introduction

Chromium is a well-known heavy metal among the various hazardous substances, which is highly toxic that can affect adversely human health. To date, it is a research hotspot to establish facile and effective methods to diminish various heavy metals from water, particularly chromium (Karimi-Maleh et al. 2021). Accordingly, an enormous variety of methods including coagulation (Qiu et al. 2019) or flocculation (Golbaz et al. 2014), active alumina/carbon adsorption (Han et al. 2019), reverse osmosis (Wu et al. 2019), ion-exchange (Nam et al. 2018), and electrodialysis (Ortega et al. 2017) have been developed to remove chromium, among which flocculation is considered as one of the best alternatives due to its cost-effectiveness advantage (Fu et al. 2017; Mahmoud et al. 2017). Flocculation has also been recognized to be an easy, low-cost, and eco-friendly approach for the aggregation of colloids, cells and suspended solids, which does not require energy input (Rossini et al. 1999). It has some advantages for industrial processes such as drinking water purification, wastewater treatment, fermentation processes, and food production because of the simplicity of liquid/solid separation (Li et al. 2016). Although many inorganic and synthetic organic flocculants have been widely applied in varied industries because of their high flocculating efficiency and cost-effectiveness, some microbial extracellular polymeric substances have been considered as a potential solution to the environmental and health problems caused by chemical flocculants (Campbell 2002; Ruden 2004).

Extracellular polymeric substances (EPS) are produced during the incubation period of microorganisms, which consist of several kinds of macromolecular polymers, such as glycoprotein, protein, polysaccharide, cellulose, and nucleic acid (Salehizadeh and Shojaosadati 2001). Thus, EPS are superior to inorganic and synthetic organic flocculants owing to their non-toxicity, biodegradability, high efficiency, free secondary pollution, and wide adaption to pH variation. Recently, EPS have been widely applied in heavy metals removal (Abu Tawila et al. 2019; Agunbiade et al. 2019; Dang et al. 2018; Fan et al. 2019), whereas reports regarding multiple applications for chromium removal from wastewater were limited. Among these EPS, the polysaccharide-based ones have been widely concerned due to their great rheological property, thermal stability, and high flocculation capacities in removing different kinds of heavy metals, dyes and pesticides from water (Abdel-Halim and Al-Deyab 2011; Crini 2005; Oliveira et al. 2020; Zhang et al. 2009). According to the previous studies, an extracellular polysaccharide from some bacterial species (*Paenibacillus polymyxa*, *Sinorhizobium meliloti* 1021, *Klebsiella* sp. J1) can remove heavy metals, including Cd (II), Pb (II), Cu (II), Zn (II), As (III), Cr (III) and Cr (VI) (Huang et al. 2019; Szewczuk-Karpisz and Wisniewska 2018; Wei et al. 2018). However, high production costs and low yields cause a bottleneck for the application of EPS to be flocculant (Smith and Miettinen 2006). As such, the improvement in the EPS production is of great importance for industrial applications.

In this study, a novel strain of *Pseudomonas fluorescens* T4 was isolated from the soil, and ARTP mutagenesis technology was applied to improve its EPS production. The EPS were then characterized and the potential applications were explored.

## Material and methods

### EPS-producing strains and culture condition

*Pseudomonas fluorescens* T4 was isolated from the soil samples collected from *Machilus Paohoi* plantation forestland in Anfu County, Jiangxi Province, China. The culture medium contained 10 g/L glucose, 1 g/L urea, 1 g/L yeast extract, 0.1 g/L K_2_HPO_4_, 0.1 g/L KH_2_PO_4_, 0.2 g/L MgSO_4_·7H_2_O, and 0.01 g/L NaCl. The *P. fluorescens* T4 was cultured in a 250-mL Erlenmeyer flask containing 50 mL culture medium and incubated on a shaking incubator (150 rpm) at 30 °C for 48 h.

### Mutagenesis by ARTP and mutant selection

The logarithmic growth phase of *P. fluorescens* T4 cells was harvested by centrifugation 8000 rpm for 10 min at 4 °C. The collected cells were washed twice with PBS buffer and diluted to a cell concentration of 10^6^ to 10^7^. Then, ARTP mutagenesis of *P. fluorescens* T4 was performed using the multi-functional plasma mutagenic system (ARTP-M, TMAXTREE Biotechnology, Wuxi, China). 10 μL cell suspension evenly scattered on the sample plate and treated with the following parameters: (i) radio frequency power input 100 W; (ii) plasma action distance 2 mm; and (iii) nitrogen gas flow rate 10 L/min. To determine the optimal treatment time, the lethality of strain T4 was investigated for different mutation durations ranging from 0 to 80 s. The processed cell suspension was cultured on agar plates at 30 °C for 48 h. The lethality rate of strain T4 was determined as follows:

1 $${\text{Lethality rate }}(\% ) = X - Y/X \times 100\%$$

where *X* is the total cell colonies without treatment, and *Y* is the total cell colonies with different mutation durations.

For screening mutation strains with enhanced flocculating activity, the appearance of the “ropy” strand colony formed on the screening plate and then mutants were inoculated into 250-mL Erlenmeyer flasks containing 50 mL fresh medium. The culture was grown in a shaker incubated for 48 h with a temperature of 30 °C and a rotational speed of 150 rpm. The flocculating activity was detected and used as an indicator for mutant selection.

### Measurement of flocculating activity

The flocculating activity was determined using the kaolin-clay suspensions model according to the previous report (Xiong et al. 2010). One milliliter of sample and 2.5 mL of CaCl_2_ (10 g/L) solution were mixed with 40 mL of 1% (wt/vol) kaolin solution, gently shaken, and incubated for 5 min at room temperature. By measuring the decrease in turbidity in the upper phase, FA was calculated using the following equation:

2 $${\text{FA }}\left( {{\text{U}}/{\text{ml}}} \right) = \left( {A--B} \right)/A \times 100 \times D$$

where *A* and *B* are the optical densities of the control and the sample at 550 nm, respectively, and *D* is the dilution factor of the cell-free culture broth. Each sample was measured in triplicate.

### Optimization of culture conditions and medium components of mutant T4-2

To optimize process parameters and the medium components, the mutant T4-2 was further investigated. Unless stated otherwise, all liquid cultures were grown in Erlenmeyer flasks (250 mL) containing medium (50 mL) and cultured on a shaking incubator (150 rpm) at 30 °C and repeated in triplicate. The culture conditions (optimum temperature, pH, and inoculum size) and medium components (carbon sources, nitrogen sources, cation, phosphate) of the cell growth and EPS production were explored.

### Batch fermentation of mutant T4-2 in a 3.6-L bioreactor

The production of EPS was investigated in a 3.6-L Benchtop bioreactor (INFORS biotechnology China Co., Ltd., Beijing, China) containing 2.0 L medium. 4% (v/v) of the seed medium which had been cultured for 16 h was inoculated into 2.0 L medium. The fermenter was controlled at 30 °C with 200 rpm of agitation rate and 2 vvm of aeration rate. Dissolved oxygen and pH are not controlled during fermentation. The fermentation was continued for about 64 h until the flocculating activity decreased. Samples were taken at regular intervals to determine the glucose concentration, bacterial biomass, flocculating activity, and EPS yield. Dissolved oxygen (DO) and pH were measured online.

### Characterization of the EPS

To remove bacterial cells, the culture broth was adjusted to pH 3.0 and centrifuged at 8000 rpm for 30 min. The obtained supernatant was adjusted to pH 7.0 and the EPS were purified by the anhydrous ethanol precipitating method as described previously (Zhen et al. 2017). The chemical elements, composition, FTIR spectroscopy, and molecular weight were characterized according to the previous report (Xiong et al. 2010).

### Jar testing for chromium (VI) removal

Chromium (VI) removal tests were carried out using different EPS concentrations (0.1–1 g/L) and various chromium (VI) concentrations (2–70 mg/L) at different solutions of pH (2.0–11.0). At a considered dose, EPS were added to 50 mL conical flasks containing 5 mL of chromium (VI) and fully mixed by shaking at 200 rpm for 20 min and then shaking at 100 rpm for 120 min in a shaker. After the adsorption step, the supernatants were filtered through a 0.45 μm pore size membrane filter. The residual chromium in the supernatant was measured by atomic absorption spectroscopy. The adsorption capacity was calculated according to the following equation:

3 $$Q\left( {{\text{mg}}/{\text{g}}} \right) = V \times \left( {C_o - C_e } \right)/W$$

where *C*_*o*_ (mg/L) and *C*_*e*_ (mg/L) are chromium (VI) concentrations in the initial and at the equilibrium, respectively, *V* (L) is the aqueous volume of the sorption reaction, *W* (g) is the mass of dry EPS, and *Q* (mg/g) is the adsorption capacity of the metal at equilibrium.

## Results and discussion

### Isolation of high flocculating activity strains from ARTP mutation

**Fig. 1:**
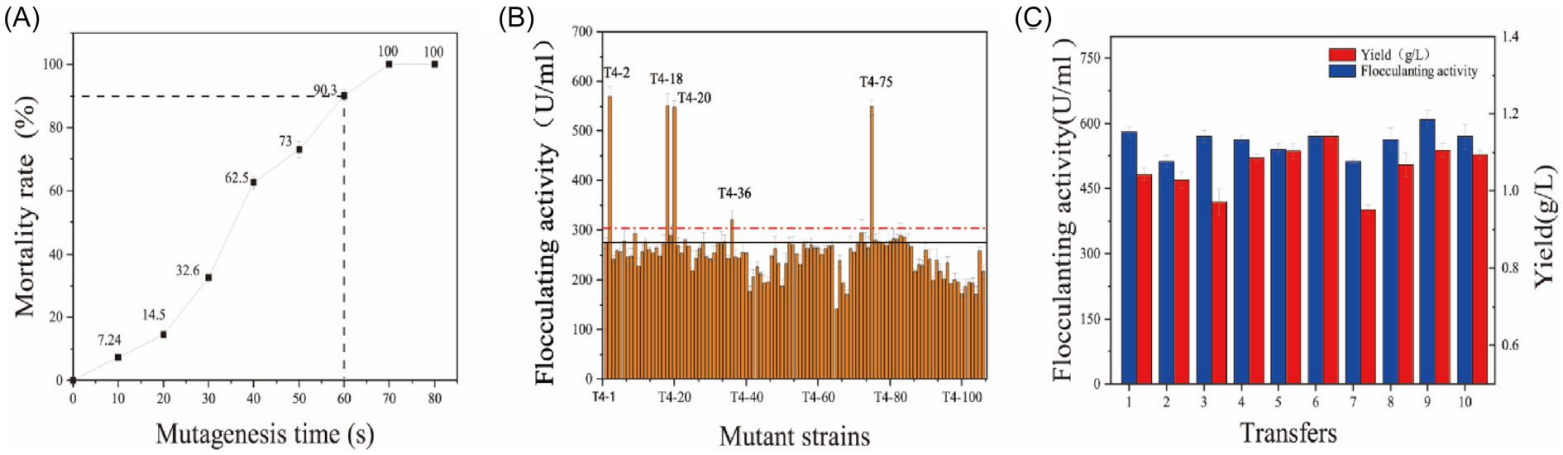
The lethality rate of strain T4 at different mutagenesis times (**A**), flocculating activity of the isolated mutants after ARTP mutagenesis (**B**) and genetic stability of mutant T4-2 in flocculating activity and EPS production (**C**)

A high cell lethality rate is desirable for efficient generation and selection of mutants. The effect of different ARTP treatment durations ranging from 10 to 100 s on the lethality rate of *P. fluorescens* is shown in Fig. 1A. According to previous reports, 90% of cell lethality percentage was set as the standard for the mutant generation (Hua et al. 2010; Liu et al. 2015). Therefore, 60 s was chosen as the appropriate exposure time in the following experiments. After plasma radiation treatment, the strain was propagated and cultured on a plate for 48 h. The mutant library was constructed from approximately 800 bacterial mutants. Developing an efficient pre-screening process to screen for desirable mutant strains is extremely crucial. The colony appearance of high-production EPS mutants showed the characteristics of “ropy” strand (Ruas-Madiedo and de los Reyes-Gavilan 2005), which can be used to screen mutants. Colonies of a total of 106 mutants of strain T4, named T4-1 to T4-106, were picked out from the mutant library using “ropy” strand, then transferred to 250-mL Erlenmeyer flasks containing 50 mL fermentation medium, and placed on a rotary shaker (150 rpm) at 30 °C, incubated for 48 h to determine the flocculating activity. Compared with the original strain, four mutants (T4-2, T4-18, T4-20, and T4-75) exhibited considerably greater flocculating activity (Fig. 1B). The highest flocculating activity (568.49 U/mL) of mutant T4-2 achieved an increase of 106.48% than the initial strain. In addition, mutant T4-2 was grown continuously to investigate its genetic stability. The mutant T4-2 exhibited the same growth and flocculating activity after several operations up to 10 rounds (Fig. 1C), reflecting that the mutant strain has favorable genetic stability in EPS production.

### Optimization of medium components and culture conditions of mutant T4-2

The secretion of microbial metabolites during fermentation depends not only on the genetic characteristics of the microorganism, but also on the composition of the medium. As previously described, there is an appreciable impact on bacterial growth and EPS yield when choosing different carbon and nitrogen sources for fermentation (Li et al. 2009; Salehizadeh and Yan 2014). It is well known that metal cations are one of the most vital factors influencing the production of EPS as it plays a crucial role in enzymatic reactions involved in EPS synthesis (Salehizadeh and Yan 2014).The results for the various carbon, nitrogen sources, and metal cations affecting the flocculating activity are described in Table 1. One noteworthy result was that mutant T4-2 grew well and produced EPS with all the carbon sources assayed. More importantly, glucose is the most efficient carbon source for bacterial growth of mutant T4-2 and EPS production. Yeast extract and urea were chosen to be the optimal complex nitrogen source for EPS production as they created the highest flocculating activity (593.7 U/mL), which is significantly higher than those with alternative nitrogen sources. As shown in Table 1, Mn^2+^ is chosen as the optimal cation for EPS production by mutant T4-2. Due to the great influence on microbial metabolism, especially the production of EPS, the C/N ratio associated with EPS production has received great attention (More et al. 2014). From this, the concentrations of glucose, urea, and yeast extract were determined, and the results are shown in Additional file 1: Figure S1. In addition, the effect of the concentration of inorganic salts and metal cations on EPS production was investigated and the results are shown in Additional file 1: Figure S2. Based on the experimental results, the optimal medium component was obtained as follows (g/L): glucose 15.0, yeast extract 0.8, urea1.0, KH_2_PO_4_ 0.1, K_2_HPO_4_ 0.1, MnCl_2_ 0.1, and NaCl 2.0.

**Table 1:** Effect of different carbon sources, nitrogen sources, and cations on the production of the EPS from *P. fluorescens* mutant T4-2

Carbon source	FA ± SD (U/mL)	OD_600_	Nitrogen source	FA ± SD (U/mL)	OD_600_	Cation	FA ± SD (U/mL)	OD_600_
Sucrose	519.5 ± 21.1	4.00 ± 0.01	Urea	36.1 ± 13.3	0.68 ± 0.04	Mg^2+^	1264.1 ± 26.4	3.56 ± 0.13
Fructose	243.5 ± 9.9	4.69 ± 0.02	NaNO3	26.5 ± 5.1	0.50 ± 0.04	Ca^2+^	1257.5 ± 15.1	4.20 ± 0.12
Glucose	583.7 ± 5.1	5.23 ± 0.06	NH4Cl	179.8 ± 35.2	3.73 ± 0.02	Mn^2+^	1285.1 ± 12.4	4.22 ± 0.09
Glycerol	26.1 ± 6.6	3.12 ± 0.12	Yeast extract	31.4 ± 4.4	1.84 ± 0.21	K^+^	623.4 ± 19.1	4.26 ± 0.02
Lactose	274.4 ± 7.3	4.38 ± 0.15	Tryptone	151.3 ± 21.7	3.00 ± 0.09	Fe^3+^	0 ± 0	3.32 ± 0.13
Maltose	259.3 ± 19.6	4.62 ± 0.04	Beef extract	201.5 ± 32.1	2.26 ± 0.15	Al^3+^	618.4 ± 20.0	4.33 ± 0.21
Xylose	256.5 ± 17.4	4.53 ± 0.04	Soy flour	56.7 ± 7.4	1.09 ± 0.14	Zn^2+^	32.5 ± 3.1	2.97 ± 0.24
Sodium citrate	229.5 ± 23.0	4.83 ± 0.04	Mixed nitrogen	593.7 ± 5.1	4.91 ± 0.09	Cu^2+^	2.7 ± 1.8	0.18 ± 0.02

FA: flocculating activity, SD: standard deviation, mixed nitrogen: [yeast extract + urea]

Culture temperature, initial media pH, and inoculum size are also important factors affecting flocculent activity and EPS production. The optimized culture conditions are shown in Additional file 1: Figure S3. The flocculating activity reached 2579.9 U/mL under these conditions, and the EPS yield reached 4.84 g/L. Besides, it was found that the cell-free supernatant possessed nearly 95% flocculating activity (2450 U/mL), while cells only displayed flocculating activity of 28 U/mL. Following the previous findings (Subudhi et al. 2016; Xia et al. 2008), the cell-free supernatant was found to have greatly higher activity than the culture pellet, indicating that the EPS produced by *P. fluorescens* mutant T4-2 have flocculating activity.

### Fermentations of *P. fluorescens* mutant T4-2 on 3.6-L bioreactor

**Fig. 2:**
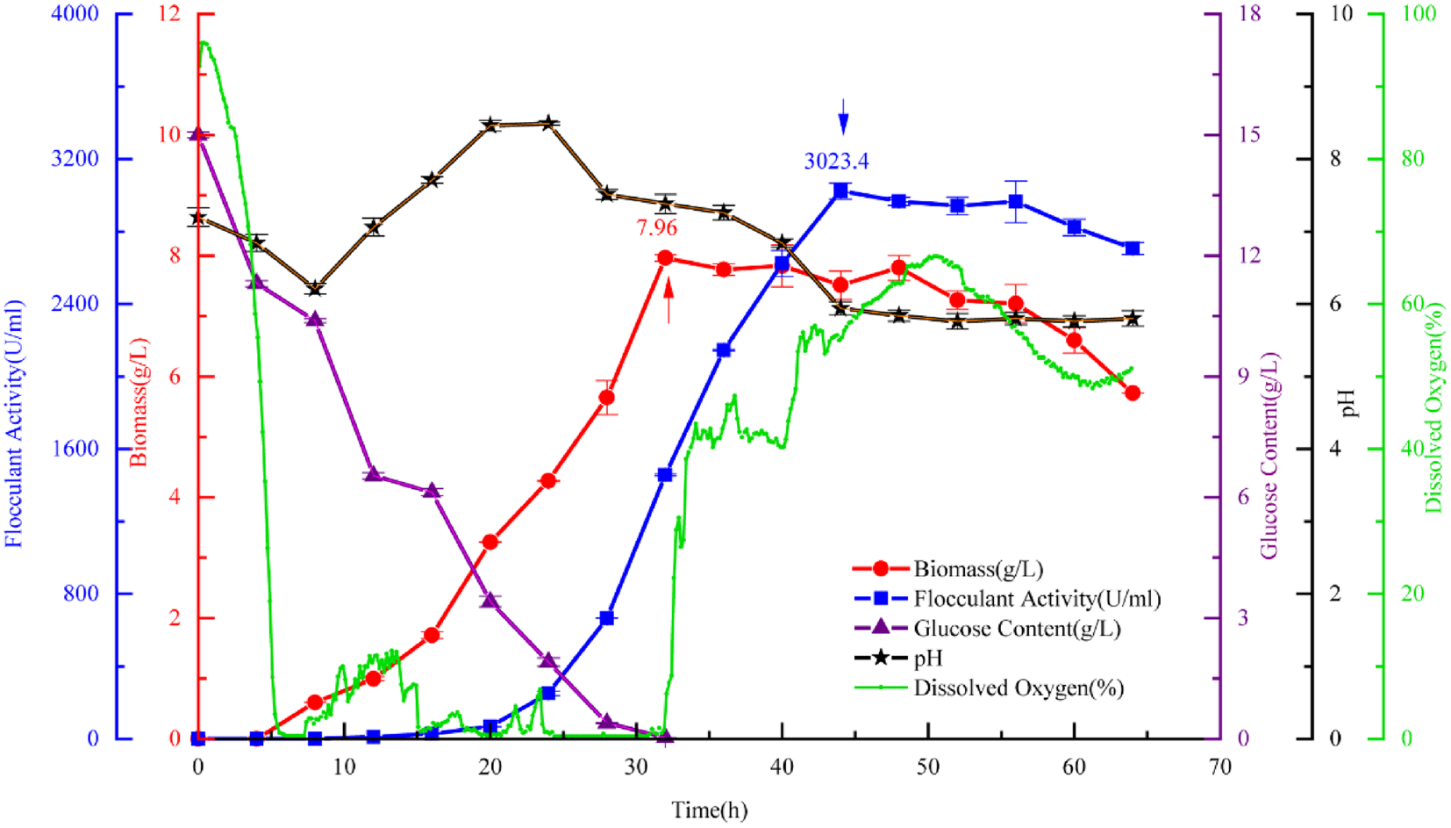
Fermentation of *P. fluorescens* mutant T4-2 in a 3.6-L bioreactor in an optimized medium

To evaluate the performance of the *P. fluorescens* mutant T4-2 under a more stable condition, batch fermentation was performed in a 3.6-L bioreactor with optimal culture media and culture conditions. As shown in Fig. 2, the flocculating activity and biomass were significantly improved. During the exponential phase, dissolved oxygen (DO) decreased rapidly to about 10% and then continued to decline to 0 at 6 h, indicating that the growth of bacteria needs large amounts of oxygen. After 28 h of fermentation, the glucose in the medium was practically exhausted and the biomass reached a maximum value of 7.96 g/L at 32 h and a maximum flocculating activity of 3023.4 U/mL at 46 h. The trend of development of both curves is consistent, but the flocculating activity curve shows a hysteresis lag compared to the biomass. When cell growth entered a stable phase, EPS still accumulated until 10 h later, with the flocculating activity reaching the highest level. During the later stages of fermentation, the flocculating activity and biomass decreased, possibly because some of the bacteria were autolyzed when nutrients were used up, and then various enzymes in the cell were exposed. The hydrolysis of the EPS by these enzymes results in the decrease in flocculating activity (Yu et al. 2016). The flocculating activity of mutant T4-2 in the 3.6-L bioreactor was 3023.4 U/mL, 17.19% higher than that in conical flasks (2579.9 U/mL). The final yield of EPS reached 6.42 g/L, 34.1% higher than that of the cultivation in conical flasks (4.84 g/L). The result is clearly higher than most EPS-producing strains, such as *Aspergillus flavus* (Aljuboori et al. 2013), *Bacillus* sp. (Okaiyeto et al. 2015), and *Proteus mirabilis* TJ-1 (Xia et al. 2008).

### Characterization of EPS

**Fig. 3:**
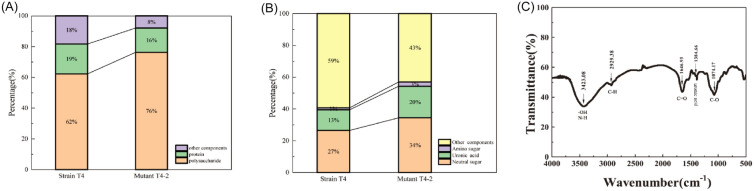
Components in EPS from *P. fluorescens* T4 and mutant T4-2 (**A**), percentage of neutral sugar, uronic acid and amino sugar in EPS of *P. fluorescens* T4 and mutant T4-2 (**B**) and infrared spectra of EPS from *P. fluorescens* mutant T4-2 (**C**)

The EPS of *P. fluorescens* mutant T4-2 were mainly composed of polysaccharide (76.27%) and protein (15.8%). Compared with the original *P. fluorescens* T4, the polysaccharide content of EPS from T4-2 increased by 14.01% and protein content decreased by 3.65% (Fig. 3A) which may be an indication that polysaccharide plays a more important role in the flocculating activity of the polymer. Besides, the neutral sugars (34.43%), uronic acids (19.84%), and amino sugars (2.71%) in a polysaccharide fraction of EPS were also improved than those of the original strain T4 (Fig. 3B). It was further observed that uronic acid and amino sugar in polysaccharide make most of the contribution to the flocculation capacity of the EPS molecule, attributing to their carboxyl groups and amide groups, which are conducive to adsorption particles and flocculation (Gao et al. 2006). Tiwari et al. (2015) also reported that there was a positive correlation between the uronic acid content and flocculating activity.

The functional groups of the EPS were identified using FT-IR spectroscopy. The result of FT-IR is shown in Fig. 3C and consistent with the previous study (Xiong et al. 2010), indicating the presence of hydroxyl, amide, and carboxyl groups in the EPS. Elemental analysis showed that EPS from *P. fluorescens* mutant T4-2 contained C, H, N, 29.64%, 5.24%, and 5.20%, respectively. The average molecular weight of the EPS was 1.17 × 10^5^ Da, which is significantly higher than other EPS reported in the literature (Aljuboori et al. 2013; Li et al. 2010, 2009). High molecular weight contributed to the flocculating activity because of extra adsorption points and stronger bridging (Giri et al. 2015; Kumar et al. 2004).

### Performance of the EPS in chromium (VI) removal

#### Effect of the solution pH

**Fig. 4:**
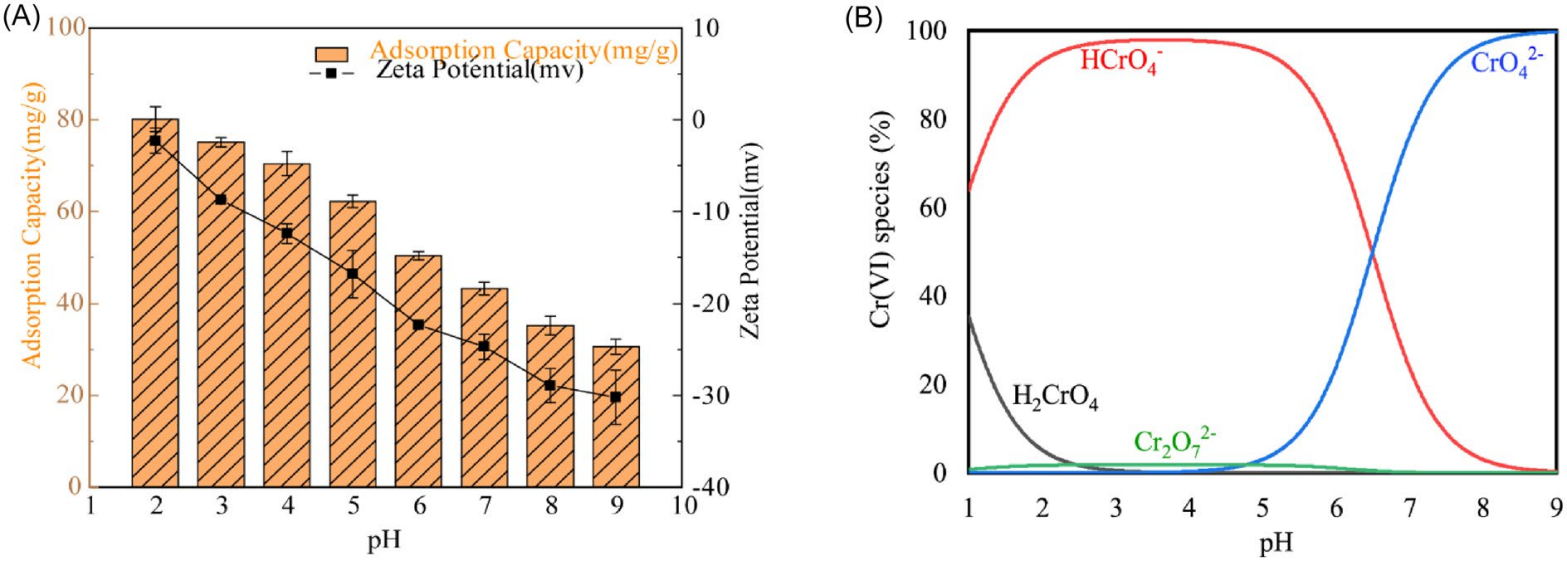
Effect of pH on the removal of the Cr (VI) and the corresponding zeta potential of EPS (**A**), species of H_2_CrO_4_, HCrO_4_^−^, Cr_2_O7^2−^ and CrO_4_^2−^ at different pH, *C*_0_ = 50.0 mg/L, *T* = 298 K (**B**)

The initial pH of the solution has a major impact on the adsorption process of heavy metal ions. It affects not only the charged surface functional group of EPS but also the form of chromium in the solution. As illustrated in Fig. 4A, the effect of pH on Cr (VI) adsorption by the EPS and the zeta potential of the EPS were investigated. The zeta potential of EPS decreases from − 2.3 to − 30.2 as the pH increases from 2 to 9, indicating that the surface charge of EPS is negatively charged. The species distribution of Cr (VI) is shown in Fig. 4B, and Cr (VI) exists by two anions of HCrO^4−^ and Cr_2_O7^2−^ in the range of pH 2–9. From Fig. 4A, it shows that the adsorption capacity of the Cr (VI) was found to decrease with the increase in the initial pH of the contact solution. However, large amounts of protons have a positive effect of promoting the reduction of Cr (VI) to Cr (III) at low pH. Cr (III) mainly exists in acid solutions in the form of three cations of Cr^3+^, CrOH^2+^, and Cr(OH)^2+^. The negatively charged EPS surface was easy to bind with the positively charged ionic groups of Cr (III) through electrostatic attraction, thus reducing the Cr (VI) anionic species in the solution. On the other hand, it is reported that amides (–NH–) on the extracellular polymer might have an effect on Cr (VI) reduction. Wei et al. (2015) used EPS from *Klebsiella* sp. J1 to remove Cr (VI) in aquatic environments, and analyzed the adsorption mechanism with Zeta-potential meter, X-ray photoelectron spectrometer (XPS), and Fourier transform infrared spectrometer. They found that 82.3% Cr (VI) was reduced to Cr (III) by amine group (–NH–) in EPS. Thus, we supposed similar reduction process with EPS from *P. fluorescens* which contains amide (–NH–) groups from FT-IR spectroscopy (Fig. 3C). Generated Cr (III) immobilize on the surface of the EPS by electric neutralization, thereby reducing the content of Cr (VI) in the solution and improving the adsorption efficiency. According to Fig. 3C, EPS not only contains amino groups, but also carboxyl and hydroxyl groups, the presence of which leads to the formation of carboxyl and amino coordination groups, as well as hydroxyl and carboxyl coordination groups. This chemical structure facilitates the chelation of metal ions. Zhou et al. (2019), Chug et al. (2016) and Pi et al. (2017) also confirmed the above conjectural result. Therefore, we conjecture that Cr (VI) is also adsorbed onto the surface and internal coordination groups of EPS by chelation during the adsorption process in this study. The adsorption of Cr (VI) by EPS decreased from 80.13 to 30.67% as the pH increased from 2 to 9. An acidic environment is preferred for effective Cr (VI) adsorption (Guo and Chen 2017), the highest adsorption capacity of Cr (VI) reached as high as 80.13 mg/g at pH 2. These results (Fig. 4) demonstrated that the EPS exhibited a high adsorption affinity toward Cr (VI).

#### Adsorption isotherm and kinetics for chromium (VI) removal

**Fig. 5:**
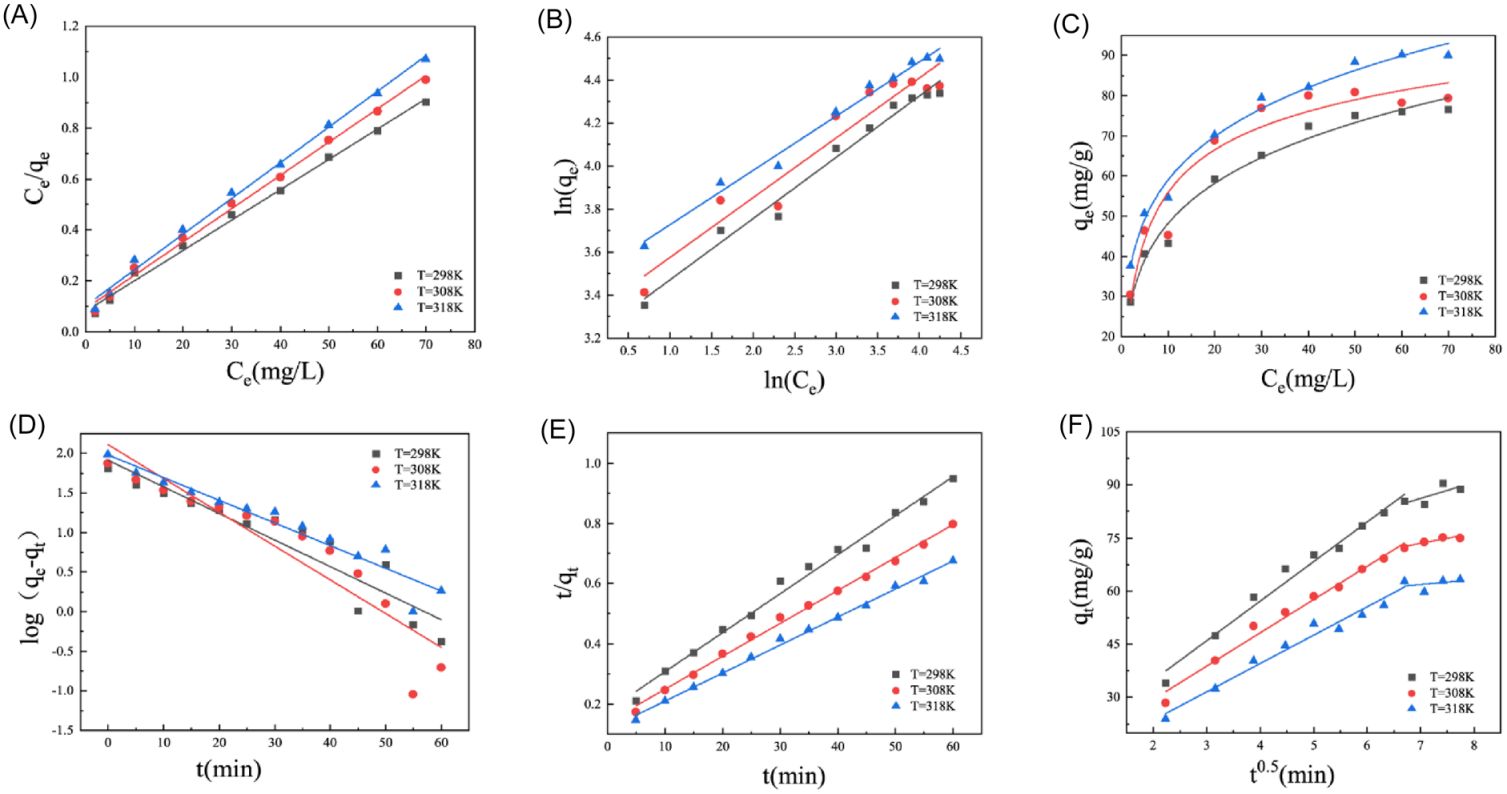
The adsorption model for chromium (VI) Langmuir model (**A**) Freundlich model (**B**) Redlich Peterson (**C**) and the adsorption kinetic model for chromium (VI) Pseudo-first-order kinetic (**D**) Pseudo-second-order kinetic (**E**) Webber–Morris kinetic (**F**)

Adsorption isotherm can reflect the distribution of adsorbed molecules in the liquid phase and solid phase in equilibrium at a certain temperature; it is an essential index to evaluate the adsorption performance of adsorbent. Adsorption models of Langmuir, Freundlich, and Redlich Peterson were studied to simulate Cr (VI) adsorption by EPS. According to the fitting results of three models to the experimental data as Fig. 5A–C showed, the parameters (*q*_m_, *B*, *K*_F_, *n*, *K*, *α*, *β*) and correlation coefficient *R*^2^ were calculated, and the calculated results are given in Table 2. The Langmuir model had the highest fitting correlation coefficient for Cr (VI) adsorption process, which implies that the Cr (VI) adsorption by EPS belongs to homogeneous adsorption, and the adsorption sites do not interfere with each other. Once the adsorption is complete, it is no longer affected by the absorbent. The analysis of EPS for removal Cr (VI) in aqueous showed that the above three adsorption isotherm models can better fit the adsorption process (*R*^2^ > 0.91). It showed that the EPS adsorption process for Cr (VI) was complex and may involve several adsorption mechanisms. This may be due to the peculiar structure of EPS, such as porous structures which can lead to multi-step adsorption processes. The parameters *q*_m_ and *B* in the Langmuir adsorption isotherm model decreased with increasing temperature, indicating that the Cr (VI) adsorption by EPS was an exothermic process.

**Table 2:** Isotherm parameters for the adsorption of chromium (VI)

	*T* (K)	Langmuir isotherm			Freundlich isotherm			Redlich–Peterson isotherm			
		*q*_m_ (mg/g)	*B* (L/mg)	*R*^2^	*K*_F_ (mg/g)	*n*	*R*^2^	*K*	*α*	*β*	*R*^2^
Cr (VI)	298	83.890	0.146	0.994	24.180	3.510	0.978	61.805	1.984	0.775	0.975
	308	76.510	0.142	0.995	27.025	3.597	0.920	27.723	0.512	0.888	0.914
	318	71.320	0.136	0.994	32.292	3.964	0.984	128.730	3.417	0.784	0.981

Adsorption kinetics is the relation between the adsorption capacity and the adsorption time, which is a crucial feature to describe the adsorption efficiency. Kinetic models of pseudo-first-order, pseudo-second-order, and Webber–Morris were used to simulate the dynamical experimental data, and the experimental results are shown in Fig. 5D–F. The premise of the pseudo-first-order kinetic model is that diffusion is assumed to be the main step in controlling the adsorption rate, whereas the pseudo-second-order kinetic model considers chemisorption between the adsorbate ion and the adsorbent active site as the main step in controlling the adsorption rate. For evaluating the type of reaction mechanism involved, according to the fitting results of the dynamical experimental data, the parameters (*k*_1_, *q*_e_, *k*_2_) and correlation coefficient *R*^2^ of each model were calculated, and the calculated results are shown in Table 3. The formulas used to calculate these parameters of the two models are given in Additional file 1: Table S1. For the adsorption of Cr (VI) by EPS, the pseudo-second-order kinetic model was a better fit than the pseudo-first-order kinetic model. The theoretical equilibrium adsorption capacity *q*_e_ of Cr (VI) is 90.90 mg/g from Table 3, which is closer to the experimentally measured equilibrium adsorption capacity of 80.13 mg/g. The results also indicate that the rate-limiting step of the adsorption process was chemical adsorption, which is consistent with the results of previous studies on the removal of heavy metal ions by flocculant (Fan et al. 2019). FT-IR analysis indicated the presence of hydroxyl, amide, and carboxyl functional groups in the EPS, which possibly provide active sites in Cr (VI) aggregations (Xu et al. 2012).

**Table 3:** Kinetic parameters for the adsorption of chromium (VI)

*T* (K)	Pseudo-first-order kinetic			Pseudo-second-order kinetic		
	*k*_1_ (min^−1^)	*q*_e_ (mg/g)	*R*^2^	*k*_2_ (mg/g/h)	*q*_e_ (mg/g)	*R*^2^
298	0.055	78.700	0.867	0.743 × 10^–3^	107.520	0.989
308	0.058	66.830	0.849	0.853 × 10^–3^	91.580	0.996
318	0.050	53.080	0.926	0.942 × 10^–3^	77.280	0.995

#### Thermodynamic parameters and activation energy

The thermodynamic parameters, namely, the Gibbs free energy change (Δ*G*^0^), standard enthalpy change (Δ*H*^0^), and standard entropy change (Δ*S*^0^), were calculated according to the Van't Hoff equation (Additional file 1: Table S1) to evaluate the adsorption thermodynamic behavior. The values of the thermodynamic parameters for Cr (VI) adsorption onto EPS are given in Table 4. The Gibbs free energy change Δ*G*^0^ was negative at various temperatures, confirming that the adsorption was spontaneous and thermodynamically favorable. The absolute value of Δ*G*^0^ gradually increased with the increasing temperature, which proved that the increasing temperature was beneficial to the adsorption process. The negative values of Δ*H*^0^ indicated that adsorption was exothermic, which agrees with the results of the Langmuir adsorption isotherm model. The positive values of the entropy Δ*S*^0^ suggest an increasing randomness during the adsorption process (Xu et al. 2012). Activation energy is the amount of energy required for a molecule to transition from a normal state to an active state where chemical reactions can easily occur. The value of the activation energy Ea was calculated as 9.36 kJ/mol from the Arrhenius equation (Additional file 1: Table S1). It affirmed that the physisorption phenomenon is the prevailing reaction because the activation energy is in the range of 5–40 kJ/mol. (Wei et al. 2015).

**Table 4:** Activation energy and thermodynamic parameters for adsorption of chromium (VI)

Ea (KJ/mol)	Δ*G*^0^ (KJ/mol)			Δ*H*^0^ (KJ/mol)	Δ*S*^0^ (J/mol/K)
	298 K	308 K	318 K		
9.360	− 21.830	− 22.210	− 22.490	− 2.787	82.750

Recently, EPS has attracted more researchers' interest because of their heavy metal removal properties (Li et al. 2022; Siddharth et al. 2021). Due to different chemical structures and functional groups, EPS from different strains have different performances in removing heavy metals. The reported adsorption of heavy metals by EPS mainly focused on the adsorption of Cd (II) (Yin et al. 2013), Cu (II) (Wang et al. 2014), Pb (II) (Kumari et al. 2017), Zn (II) (Li et al. 2019), and Ni (II) (Nkoh et al. 2019), but there are few reports on the adsorption of Cr (VI). The EPS from *Pseudomonas fluorescens* in this study are better than most reported in the adsorption of Cr (VI) (Paul et al. 2012; Pi et al. 2021). The adsorption process is a popular, widely adopted and efficient process for removing Cr (VI) from aqueous solutions. As an environmentally friendly and easily degradable Cr (VI) adsorbent, EPS in this study is an adsorbent with potential industrial application potential whether it is used directly or combined with other flocculants. However, these EPS for other heavy metals removal are still under investigation.

## Conclusion

ARTP was adopted to obtain a *Pseudomonas fluorescens* mutant T4-2 with higher flocculating activity and higher EPS yield. With the optimized cultivation conditions, EPS yield from T4-2 was increased to 6.42 g/L with a flocculating activity of 3023.4 U/mL on a 3.6-L bioreactor. Its efficiency for Cr (VI) removal was also proved. The adsorption capacity for Cr (VI) was determined to be 30.66–80.13 mg/g in the pH range from 2 to 9 and the removal efficiency of Cr (VI) solution reached as high as over 80%. The adsorption mechanism was further speculated according to the Langmuir isotherm and pseudo-second-order kinetic models, which proposed that the chemical adsorption may be the rate-limiting step and physical adsorption dominates the process. The high production and Cr (VI) removal efficiency suggested great industrial potential of *Pseudomonas fluorescens* EPS.

### Supplementary Information

**Additional file 1: Figure S1.** Showing the effect of different concentrations of glucose, urea, and yeast extraction EPS production from *P. fluorescens* mutant T4-2. **Figure S2.** Showing the effect of different concentrations of phosphate, Mn^2+^ and NaCl on EPS production from *P. fluorescens* mutant T4-2. **Figure S3.** Showing the optimization of culture conditions for EPS production. **Table S1.** Showing the models and equations used for the adsorption Cr(VI) of by EPS of *P. fluorescens* mutant T4-2.

## Data Availability

All data supporting the findings of this study are available in the article, supporting information, or upon request from the corresponding author.

## Abbreviations

ARTP: Atmospheric and room temperature plasma
EPS: Extracellular polymeric substances
NCBI: National Center for Biotechnology Information
