# Supplementary material for: Hyperproduction of extracellular polymeric substance in Pseudomonas fluorescens for efficient chromium (VI) absorption
Source: Bioresour Bioprocess. 2023 Mar 8;10(1):17. doi: 10.1186/s40643-023-00638-3 (PMC10992911; doi:10.1186/s40643-023-00638-3)
Supplement: Supplementary file 1 — Additional file 1: Figure S1. Showing the effect of different concentrations of glucose, urea, and yeast extraction EPS production from P. fluorescens mutant T4-2. Figure S2. Showing the effect of different concentrations of phosphate, Mn2+ and NaCl on EPS production from P. fluorescens mutant T4-2. Figure S3. Showing the optimization of culture conditions for EPS production. Table S1. Showing the models and equations used for the adsorption Cr(VI) of by EPS of P. fluorescens mutant T4-2. [file 40643_2023_638_MOESM1_ESM.docx]

**Supplementary material**

**Hyperproduction of** **extracellular polymeric substance in *Pseudomonas fluorescens* for efficient Chromium (Ⅵ) absorption**

**Lijie Yang^1, 5^, Zhen** **Chen^3^, Ying Zhang^2^, Fuping Lu^4^, Yihan Liu^4*^, Mingfeng Cao^1, 5^, Ning He^1,5*^**

^1^Department of Chemical and Biochemical Engineering, College of Chemistry and Chemical Engineering, Xiamen University, Xiamen 361005, P. R. China

^2^Shandong Institute of Commerce and Technology, Jinan 251000, P.R. China

^3^College of Life Science, Xinyang Normal University, Xinyang 464000, P. R. China

^4^Key Laboratory of Industrial Fermentation Microbiology, Ministry of Education, Tianjin Key Laboratory of Industrial Microbiology, The College of Biotechnology, Tianjin University of Science and Technology, Tianjin 300457, China

^5^The key Lab for Synthetic Biotechnology of Xiamen City, Xiamen University, Xiamen 361005, P. R. China

***Correspondence****:** Ning He, Yihan Liu

E-mail address: [hening@xmu.edu.cn](mailto:hening@xmu.edu.cn), [lyh@tust.edu.cn](mailto:lyh@tust.edu.cn)

Tel.: +86-592-2183751

Fax: +86-592-2184822


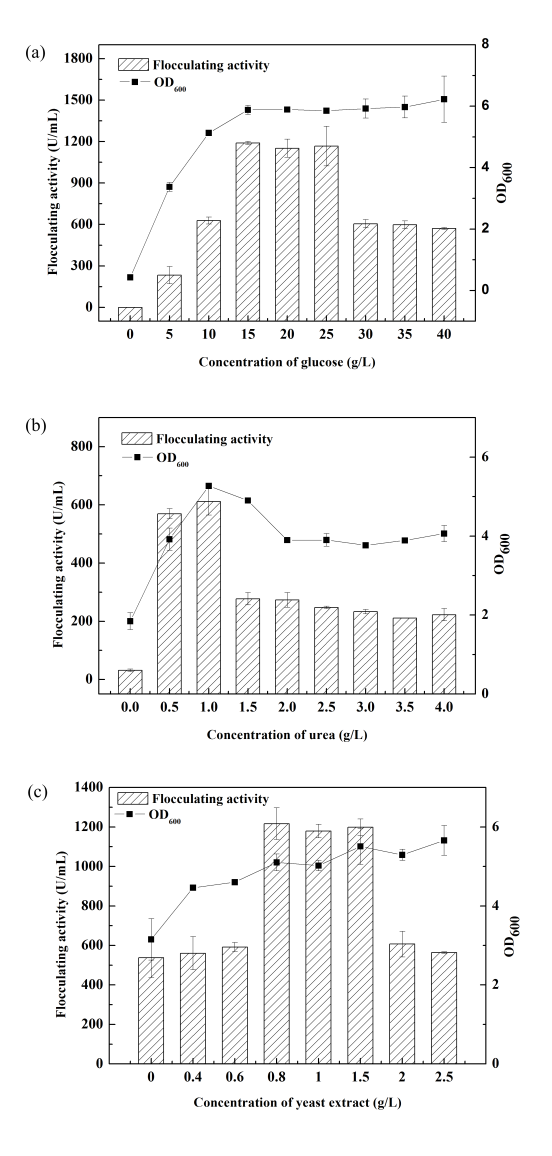


Figure S1 Effect of different concentration of glucose (a), urea (b) and yeast extract (c) on EPS production.


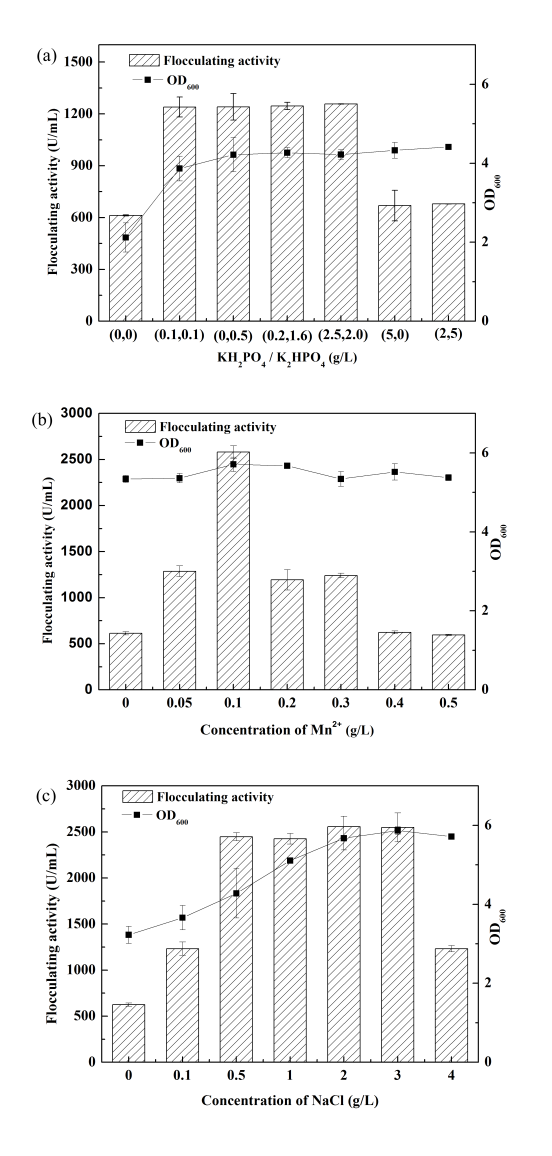


Figure S2 Effect of different concentration of phosphate (a), Mn^2+^ (b) and NaCl (c) on EPS production.


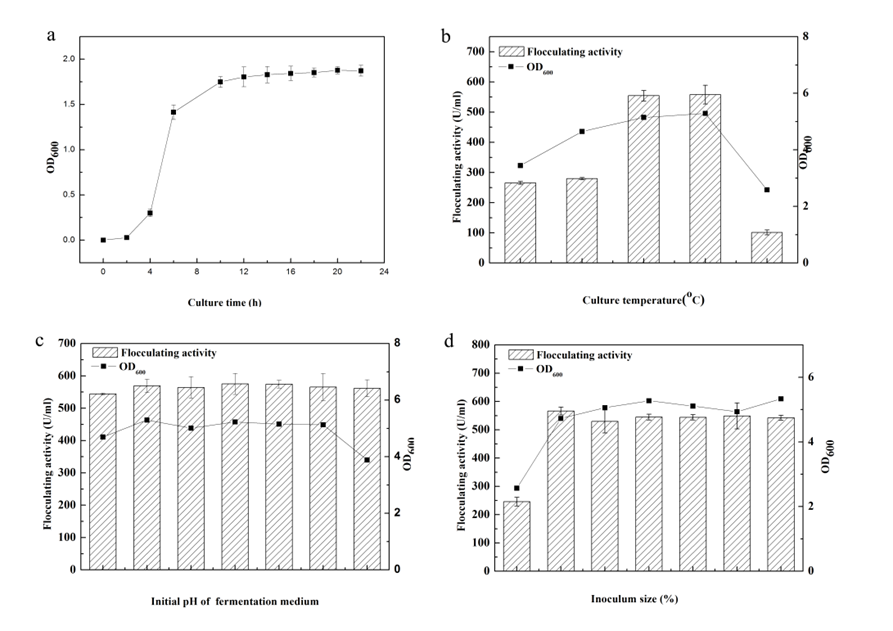


Figure S3 Optimization of culture conditions for EPS production. (a) Growth curves of *P. fluorescens* mutant T4-2; Effect of temperature (b), initial pH (c) and inoculums size (d) on EPS production.

Table S1. The models and equations used for the adsorption Cr (VI) of by EPS of *P. fluorescens* mutant T4-2

|  | Model | Equation | Model parameters |
| --- | --- | --- | --- |
| Adsorption isotherm | Langmuir | $\frac{1}{q_{e}}=\frac{1}{q_{m}}+\frac{1}{bq_{m}C_{e}}$ | q_e_ = equilibrium capacity of Cr (VI) adsorbed onto EPS (mg g^−1^), C_e_ = equilibrium concentration of Cr (VI) solution (mg L^−1^), q_m_ = maximum adsorption capacity (mg g^−1^), and b = energy of adsorption (L mg^−1^); |
|  | Freundlich | $\log q_{e}$=$\log K_{F}+\frac{1}{n}\log C_{e}$ | q_e_ = equilibrium capacity of Cr (VI) adsorbed onto EPS (mg g^−1^), C_e_ = equilibrium concentration of Cr (VI) solution (mg L^−1^), K_F_ = Freundlich constant (mg g^-1^), n = adsorption intensity of the EPS |
|  | Redlich-Peterson | $q_{e}=\frac{KC_{e}}{1+\alpha C_{e}^{\beta}}$ | K and α are the Redlich−Peterson isotherm constants and β is the exponent |
| Adsorption kinetics | Pseudo-first order | $\log(q_{e}-q_{t})=\log q_{e}-\frac{k_{1}}{2.303}$t | q_e_ = equilibrium adsorption capacity (mg g^−1^), q_t_ = adsorption capacity (mg g^−1^) at any time t, k_1_ = first-order rate constant(h^-1^) |
|  | Pseudo-second order | $\frac{t}{q_{t}}=\frac{1}{k_{2}{q_{e}}^{2}}+\frac{1}{q_{e}}t$ | q_e_ = equilibrium adsorption capacity (mg g^−1^), q_t_ = adsorption capacity (mg g^−1^) at any time t, k_2_ = the second-order rate constant (mg g^−1^ h^−1^) |
|  | Webber-Morris | $q_{t=k_{i}t^{0.5}+c}$ | q_t_ = adsorption (mg g^−1^) at any time t, k_i_ = intra-particle diffusion rate constant (mg g^−1^ h^−0.5^) |
| Adsorption thermodynamics | Vant Hoff’s equation | $\Delta G^{0}=\Delta H^{0}-T\Delta S^{0}$  $\Delta G^{0}=-RTlnK_{0}$  $lnK_{0}=\frac{\Delta S^{0}}{R}-\frac{\Delta H^{0}}{RT}$ | ΔG^0^= Gibbs free energy change (kJ mol^−1^), R = the gas constant (J mol^−1^ K^−1^), T = temperature (K), k_0_ = the equilibrium constant, ΔH^0^ = enthalpy change (kJ mol^−1^), ΔS^0^ = entropy change (kJ mol^−1^ K^−1^) |
|  | Arrhenius equation | $lnK=lnA-\frac{E_{a}}{RT}$ | *E_a_* =the Arrhenius activation energy (kJ mol^−1^), *A* = the Arrhenius factor, *R*= the Arrhenius factor, *K* = the rate constant (*k*2) of the pseudo-second order kinetic mode |
